# The Construction of Impossibility: A Logic-Based Analysis of Conjuring Tricks

**DOI:** 10.3389/fpsyg.2016.00748

**Published:** 2016-06-14

**Authors:** Wally Smith, Frank Dignum, Liz Sonenberg

**Affiliations:** ^1^Department of Computing and Information Systems, The University of MelbourneMelbourne, VIC, Australia; ^2^Department of Information and Computing Sciences, Universiteit UtrechtUtrecht, Netherlands

**Keywords:** stage magic, conjuring, propositional logic, impossibility

## Abstract

Psychologists and cognitive scientists have long drawn insights and evidence from stage magic about human perceptual and attentional errors. We present a complementary analysis of conjuring tricks that seeks to understand the experience of impossibility that they produce. Our account is first motivated by insights about the constructional aspects of conjuring drawn from magicians' instructional texts. A view is then presented of the logical nature of impossibility as an unresolvable contradiction between a perception-supported belief about a situation and a memory-supported expectation. We argue that this condition of impossibility is constructed not simply through misperceptions and misattentions, but rather it is an outcome of a trick's whole structure of events. This structure is conceptualized as two parallel event sequences: an *effect sequence* that the spectator is intended to believe; and a *method sequence* that the magician understands as happening. We illustrate the value of this approach through an analysis of a simple close-up trick, Martin Gardner's *Turnabout*. A formalism called propositional dynamic logic is used to describe some of its logical aspects. This elucidates the nature and importance of the relationship between a trick's effect sequence and its method sequence, characterized by the careful arrangement of four evidence relationships: similarity, perceptual equivalence, structural equivalence, and congruence. The analysis further identifies two characteristics of magical apparatus that enable the construction of apparent impossibility: substitutable elements and stable occlusion.

## Introduction

The methods of stage magicians have long been regarded as a potential source of insight into the workings of the human mind. Around the turn of the nineteenth century, several leading figures in the new psychological sciences extended an interest in visual illusions to the illusions of stage magic (e.g., Binet, 1894; Jastrow, 1900; Triplett, 1900). Connections between magic and psychology have been made periodically since then (e.g., Kelley, 1980; Hyman, 1989), including links to cognitive science (Kuhn et al., 2008) and cognitive neuroscience (e.g., Macknik et al., 2008; Parris et al., 2009; Leeuwen, 2011). The premise underlying all of these investigations is that conjuring tricks, that routinely and reliably bring about radical failures in how people make sense of the world, might open a new window into how that sense is normally achieved.

Many of these investigations have focussed on understanding localized points of perceptual or attentional failure within the performance of a magic trick (e.g., Cui et al., 2011; Kuhn and Martinez, 2011). In this paper, we seek to complement this line of research by exploring a parallel question of how spectators reach an experience of witnessing something impossible. This requires a different kind of explanation to that for how misperceptions and misattentions occur. In the course of normal life, people frequently misperceive or misattend relevant events but this almost never produces the dramatic experiences of impossibility that characterize successful magic tricks. Rather, people typically discount everyday anomalies in their sense-making through metacognitive awareness of the fallibility of their perceptual, attentional and cognitive systems. The question arises, then, as to how it is that a spectator of a trick, who has also misperceived or misattended events, does not simply discount the final magical effect because they aware are that sensory information and therefore sense-making is fallible. To reach its conclusion, a magic trick must be designed and performed not only to deceive perception and attention, but also to trap the human mind in a situation where the only sense that can be made is of something impossible having occurred.

In this article, we attempt to develop an account of the logical form of beliefs that a spectator of a conjuring trick holds to underpin the experience of witnessing an impossible event. In this way, we seek to add to recent mathematically-based treatments of magic more generally, both in the workings of tricks (e.g., Diaconis and Graham, 2011) and in theorizing about their computational aspects (e.g., Williams and McOwan, 2014). Our aim is to show that the precision in expression mandated by the demands of assigning meaning to the components of logical formalisms can serve to illuminate the underlying complexity of beliefs that underpin even a simple conjuring trick. This complements other logical and computational treatments of related experiences such as surprise (e.g., Ortony and Partridge, 1987; Casati and Pasquinelli, 2007; Lorini and Castelfranchi, 2007; Macedo et al., 2009), as well as accounts of surprise from mathematical (Baldi and Itti, 2010) and psychological (Maguire et al., 2011) perspectives. In these studies, surprise is generally regarded as a belief-based phenomenon, associated with disconfirmed expectations. Some approaches have considered how an event is processed, represented, and integrated within an unfolding scenario theorized as a sequence of world states, successively changing by the application of actions (e.g., Maguire et al., 2011). We adopt a similar approach to the understanding of impossibility.

An important premise of our analysis is that to understand how an experience of impossibility is reached demands an understanding of the full sequence of a trick's events. Kelley (1980) took a similar approach in a qualitative analysis of magic tricks from the perspective of attribution theory. For a particular card trick, the “Whispering Queen,” he mapped out its structure in terms of an “apparent causal sequence” in seven steps, of what the spectator perceives, against the corresponding events of a “real causal sequence.” It was discrepancies between the two sequences seen as a whole that resulted in the experience of an “extraordinary or supernatural cause-effect” relation. Our aim is to take the essence of Kelley's approach further, albeit with different terms and concepts, and thereby to focus on what we will refer to as the *constructional* aspects of conjuring tricks. As with Kelley, we consider how a trick's events are organized, as distinct from the affective aspects of the story that they project. This focus on event structure rather than story meaning resembles work in the field of narratology that studies the event structures of all narrative forms, including literature, drama and film (e.g., Landa and Onega, 2014). This is not to deny the importance of the affective aspects of conjuring, as argued by a long line of insightful magicians including Sharpe (1932), Nelms (1969) and Burger and Neale (2009). Rather, our premise is that we can independently and usefully analyse the underlying structure and logic of event sequences that create apparently impossible outcomes. This entails not just misperceived and misattended events, but the larger sequence of false and genuine actions and objects that make up a trick's performance. By implication, we focus not only on perceptual and attentional errors, but also on veridical cognitions and the metacognitive aspects of what agents believe about their beliefs and percepts. In this way, we hope to contribute to recent approaches that seek broader theories of conjuring across a range of cognitive aspects (Kuhn et al., 2014; Rensink and Kuhn, 2015).

As our starting point, the next section draws insights from magicians' texts about the constructional aspects of tricks. Following this, we develop some logical formalisms that express a general account of how an impossible situation comes about through a magic trick. To illustrate the concepts in action and to explore them further, a particular trick is then analyzed: Martin Gardner's *Turnabout* (Fulves, 1977, p. 88). It is important to emphasize that our treatment does not attempt to do justice to the full richness of the conjuror's craft. Instead we concentrate on the structure of a very simple trick with a single effect, and do not address the higher-level aspects of conjuring like routining, effect repetition, double-bluffs and false exposés; these latter things now familiar through performers such as Penn and Teller, and Derren Brown. Nevertheless we contend that important principles can be extracted from the simplest forms of conjuring. The article concludes with comments on the insights gained and the issues arising from our analysis.

## Insights from magicians' texts about the constructional aspects of conjuring tricks

The seminal writings of magicians about their craft contain a central core of ideas and principles about the way conjuring tricks should be constructed to be effective. We will briefly review these ideas from the emergence of the modern style of conjuring in the middle of nineteenth century onwards (Smith, 2015). This starts with the writings of the great French magician Jean Eugène Robert-Houdin, especially his two most famous instructional books: *Les secrets de la prestidigitation et de la magie* (Robert-Houdin, 1868) and *Magie et Physique Amusante* (Robert-Houdin, 1877). Robert-Houdin practiced and espoused a style of performance in which actions and objects were presented as being somehow natural, and it was ensured that apparatus and events were seen clearly and readily followed by audiences. The great British magician David Devant and Neville Maskelyne, of the famous Maskelyne family of conjurors, confirmed this approach in even stronger terms and in greater detail in their book *Our Magic* published in 1911. Also highly significant are the later writings of Sharpe (1932, and many others) who promoted greater dramatic meaning in conjuring effects. An American magician, Dariel Fitzkee, later popularized and extended many of the ideas in from these earlier works in an influential trilogy, including *The Trick Brain* (Fitzkee, 1944) and *Magic and Misdirection* (Fitzkee, 1945). As the popularity of stage magic declined from the 1920s onwards, new voices emerged in conjuring theory and practice from the realm of close-up magic performed for small gatherings of spectators. Highly influential are the thinking of the great Canadian-born Dai Vernon and the Argentinian-born Slydini, documented respectively by the magicians Ganson (1957) and Fulves (1976). Vernon's appeal to naturalness is firmly in the lineage of Robert-Houdin, and Maskelyne and Devant. Many general instructional texts on magic tricks have incorporated general reflections on the craft and so are relevant to this analysis. Here our selection of writings is more arbitrary but includes insights from notable magicians Jean Hugard and Harry Lorayne. In 1999, Peter Lamont and Richard Wiseman provided a concise and insightful account for non-magicians of many of these ideas and techniques, and this is also drawn on here. In recent years, a number of new significant works dedicated to the theory of conjuring have appeared that confirm many of the traditional tenets of the modern style of conjuring, while also challenging aspects and adding important new perspectives. From these we draw on Eugene Burger and Robert Neale's *Magic and Meaning* (Burger and Neale, 2009), Tommy Wonder and Stephen Minch's (Wonder and Minch, 1996) *The Books of Wonder*, and Darwin Ortiz's *Strong Magic* (Ortiz, 1994).

### Magic tricks as impossible state transitions

An important starting point for our account is to see the effect of a magic trick as an impossible state transition in which a situation passes impossibly from one state to another. We focus on tricks that fit this conception, describing them as *happenings*. In happenings, there is nothing intrinsically impossible, nor even anomalous, about the final state of objects on display (e.g., the non-existence of a coin in a purse, or the existence of a ball under a cup). Rather, the impossibility lies in how the present situation came about from the immediate history of witnessed events. This contrasts with other tricks, that might be called *spectacles*, which take the form of impossible situations presented for extended viewing (e.g., the levitation of a human body, the display of a person cut in two separated halves, or the display of a playing card as impossibly twisted so that its top and bottom face in different directions). Kelley (1980) drew a similar distinction in his account, referring to happenings as “violations of cause-effect expectations” and spectacles as “violations of entity properties.”

A state transition approach resonates with the writings of many conjuring theorists: in “any magical feat … something or somebody is caused to pass mysteriously from one place or condition to another” (Maskelyne and Devant, 1911, p. 43). Many attempts to define a taxonomy of the effects of stage magic (e.g., Sharpe, 1932; Fitzkee, 1944; Lamont and Wiseman, 1999) reflect a state transition view. For example, Sharpe's “magical plots” distinguished seven classes in which the first four illustrate a strong state transition perspective: “1. *Productions* (from not being to being)” such as producing a coin from nowhere; “2. *Disappearances* (from being to not being)” such as making the coin disappear again; “3. *Transformations* (from being in this way to being in that way)” including changes in an object with respect to its color, size, number, shape, weight; and, “4. *Transpositions* (from being here to being there)” such as making a coin jump magically from the magician's hand to being under a previously empty cup.

In addition to our focus on happenings rather than spectacles, we also focus on tricks that are strictly *impossible* (e.g., the sudden transformation of the queen of diamonds into the three of spades) as opposed to those that are highly *improbable* but strictly possible by chance (e.g., a thought-of-card later being chosen at random by a spectator). By concentrating on impossible happenings, we put emphasis on the logical and constructional aspects of magic tricks and avoid the complication of mixing logic and probability (Teigen et al., 2013).

### The principle of naturalness

Having taken a view of magic effects as impossible state transitions, we will now identify some generally accepted ideas or principles of performance that concern the constructional aspects of trick design. Perhaps the overriding principle of modern conjuring since Robert-Houdin is the idea of presenting actions and events as being *natural* (e.g., Smith, 2015), a notion that still permeates most conjuring texts. Fulves (1976, p. 14), discussing the great close-up magician Slydini, wrote: “The situation must appear natural, exactly as it would if no secret moves were performed”; and later, “Naturalness is an anesthetic to attention” (Fulves, 1976, p. 94). This points to the importance of the metacognitive aspects of deception: “The first thing that is learned is that deception depends entirely upon doing things in such a manner that it seems there is no attempt at deception” (John Scarne, attributed by Fitzkee, 1945, p. 224). Although an over-emphasis on naturalness has been criticized as potentially leading to mundane performance (Sharpe, 1932; Burger and Neale, 2009), it nevertheless persists as perhaps the most general principle of conjuring performance.

### The principle of the whole

Alongside naturalness, another key principle is that the production of impossible effects depends on the entire sequence of a trick's events, not just the faked or false actions and objects. This is a key premise of the present account, and to make it explicit we will describe it as the *principle of the whole*, although it is typically not given a name. The idea is expressed clearly by Maskelyne and Devant who saw every part of a trick as working in relation with the other parts to produce the effect, and that any unnecessary elements should be removed for artistic purity. A trick should contain “nothing beyond one continuous chain of essential details, leading to one definite effect” (Maskelyne and Devant, 1911, p. 22).

As described by Sharpe, the events of magic tricks can be divided into two parts. First is the typically longer “complication” or “preparation” phase in which apparatus is showed and displayed, elements are moved into readiness, and the procedure is explained. Second is the typically sudden “climax” when an impossible magical event is seen to have taken place. As noted by Fulves (1976, p. 17), the preparation must follow a purpose in leading to the climax, “…handling the spectator in such a way that he is first made to recognize the impossibility of what the magician is attempting; then he witnesses the dramatic realization of the impossible.”

Both parts of the trick, preparation and climax, typically include a seamless mix of genuine and false objects and actions; the magician “cleverly, skillfully, and dexterously mixes the true with the false” (Fitzkee, 1944, p. 34). The critical point is that the situation *as a whole* becomes discrepant from the spectator's understanding of it, as soon as at least one false object has been brought into play or one false action taken. This discrepancy often exists from the outset of the trick or from early on in the procedure. Once the situation is discrepant from the spectator's beliefs, even genuine objects and genuine actions become deceptive, because their implications for the situation as a whole is other than it seems. Fitzkee wrote: “the performer should be particularly careful that his handling of all of his properties, *in every respect*, is in keeping with what they are purported to be, *at all times*” (Fitzkee, 1944, p. 94; original emphasis). Hence we see throughout magic instruction great emphasis on what is often called presentation: “…remember that sleights are merely a means to an end … Unless they are surrounded by proper presentations and routines, they are worthless” (Lorayne, 1976, p. ix); and “This naturalness must not be used in a narrow sense, but also in a general sense; it must be used in everything … not only in the sleights, but in everything you do” (Dai Vernon, reported in Ganson, 1957, p. 34).

### The principle of clarity

What is essential to the modern style of conjuring since Robert-Houdin, is that the events of the preparation must be clear and readily comprehended by spectators. “The Preparation is to be made deliberately so that there is no chance of the audience missing or forgetting an incident” wrote Sharpe (1932, p. 54). Sharpe's vital point is that at the magic climax of a trick, the spectator must hold a sufficiently clear memory of the events that they believe did, and did not, happen. As Sharpe further indicated: “To do this needs considerable artistic skill in construction” (Sharpe, 1932, pp. 51/52).

Maskelyne and Devant (1911) proposed several rules of performance, many of which explicitly promote clarity: “avoid complexity” and “each effect is clear and distinct.” Fitzkee (1944, p. 34) confirmed this view: “All is built upon an unshakable foundation of naturalness, plausibility, and conviction. Here is the real skill! Here are the genuine secrets!” Vernon echoed the principle in his fundamental rules of magic: “Avoid confusion at all cost” (quoted in Cervon, 1988, p. iii). In a more specific statement, Simon (1952, p. 23) paid the following tribute to the conjuror Francis Carlyle: “One of the main reasons for his success is that he emphasizes, re-emphasizes, and over-emphasizes his effects. When he performs, there can be no doubt as to what the effect is: what has occurred. He makes his effects clear-cut, straightforward, and positively certain. If he changes a red card into a black card, you can be sure that everyone is fully aware of what the card was before the change, and what the card has changed to ….”. Again, this principle is carried forward by today's magicians: “In effects like ‘Three-Card Monte’ and the ‘Shell Game’ the audience has to try to keep track of the winning card or the pea … If you were to shift the props around so rapidly or so extensively that it required real concentration to keep track, the effect would certainly fail” (Ortiz, 1994, p. 35).

### The principle of focus

Working in tandem with the aim for clarity is the principle of focus, referring to the way that objects and actions move in and out of focal attention as the trick proceeds. While the term “misdirection” is widely used by magicians, and the wider public, most conjuring theorists have preferred to talk about the way spectators are actively directed to attend to parts of the procedure. This is not only to prevent detection of false objects and actions but also to ensure that things are generally clear: “While the magician must use all his art to disguise and cover up what he does not require to be seen, he is equally bound to make sure that every moment and every detail that ought to be seen *shall* be seen” (Maskelyne and Devant, 1911, p. 122). The Dutch conjuror Tommy Wonder (Wonder and Minch 1996, p. 13) indicated how control of focus relates to the *principles of clarity and of the whole*: “When we perform as magicians, our job consists of more than simply hiding the secret. That is just a small part of our objective. Much more important is that we highlight the important details, those things that are necessary if the audience is to understand and follow the action and its intended meaning”. An important point here is that spectators are influenced through indirect “invited inferences” (Hyman, 1989) rather than direct assertions which elicit suspicion. For example, “direct repudiation,” stating explicitly that some object or action is “normal,” is universally condemned (e.g., Maskelyne and Devant, 1911, p. 130). “Implication is always stronger than a direct statement” wrote Fitzkee (1944, p. 97).

### The principle of the incidental

Allied to controlling the focus of attention, is the manipulation of what appears necessary to the trick's plot and what is incidental. Sawing a box in two is necessary; passing the saw from one hand to the other is incidental. When performing covers for secret sleights or actions, a key technique is to choreograph them as incidental stepping stones between the supposedly more pivotal elements of the procedure. Hugard and Braue (1940, p. 444) described “the importance of the inconsequential”: “never place too much importance in your sleights, lest you telegraph to the onlookers that the sleight is about to take place.” …“The rule, subject to exception to which all rules are subject, is to treat as unimportant that which you really wish to conceal” (Hugard and Braue, 1940, p. 445). Lorayne (1976, p. ix) put it: “I have used the words ‘nonchalant’ and the phrase, ‘without hesitation,’ to the point of redundancy in this book.” Vernon (quoted in Ganson, 1957, p. 32) described how “a sleight should be a secret thing, unheralded, unhurried and unseen.”

A major challenge of trick construction is how to make a sleight or a cover for a secret action appear natural when it is contrived to work toward the impossible outcome. One technique is to manufacture the necessity for the action through a “ruse” (Fitzkee, 1945). This implies setting up a sub-goal in the plot and performance of the trick which renders the cover for the secret action as being an incidental part of a necessary sub-plot. Examples of ruses are offering an object for inspection by the audience, or picking up a wand as a tool to poke around inside a hat to show it is empty. It is in the incidental activity around these sub-routines that secret actions often lie.

### The principle of “blurring perception and inference”

A further principle which bears on how a sense of impossibility is constructed concerns how the events of a trick's history, that are partly or wholly inferred to have taken place, may later be recalled as having been perceived directly. In practice, much of the spectator's understanding of the situation is maintained through inferences about partially obscured states, like upside cards or balls under cups. During memory of the procedure, and even during its perception, spectators may not be fully aware of the boundary between the perceptual and inferential basis of their beliefs. Fitzkee (1945, p. 73) describes a trick where a money bill is placed in an envelope which is burned: “Rarely, if ever, do the spectators realize that they haven't actually seen the banknote burned.” He elaborates: “The mind has a way of putting together clues from here and there … It is an automatic process, the specific details of which the spectator is totally unaware” (Fitzkee, pp. 82/83).

### The principle of no-notice and the principle of early denial

There are many other more specific principles of trick construction. One example is the rule never to give advanced notice to the spectator of how the trick will end, or to repeat the same trick on the same occasion (e.g., Robert-Houdin, 1868; Maskelyne and Devant, 1911). To do either of these, gives the spectator too much guidance on what to scrutinize closely during the preparation stage. Another minor principle is that the procedure must be designed to quickly deny or at least contain possible explanations for the trick. During the preparation phase of the trick, actions should attempt to rule out explanations before they become well-formed suspicions: “Also it is evident that the spectators might get the idea that the banknote was ‘planted.’ So the performer takes care of this situation ahead of time” (Fitzkee, 1945, p. 56). These pre-emptive strikes must deflect not only suspicions about the genuine method of the trick, but also other possible explanations: “even wrong theories must be ruled out of spectators' minds” (Sharpe, 1932, p. 74).

## A formal analysis of the construction of impossibility

**Figure 1:**
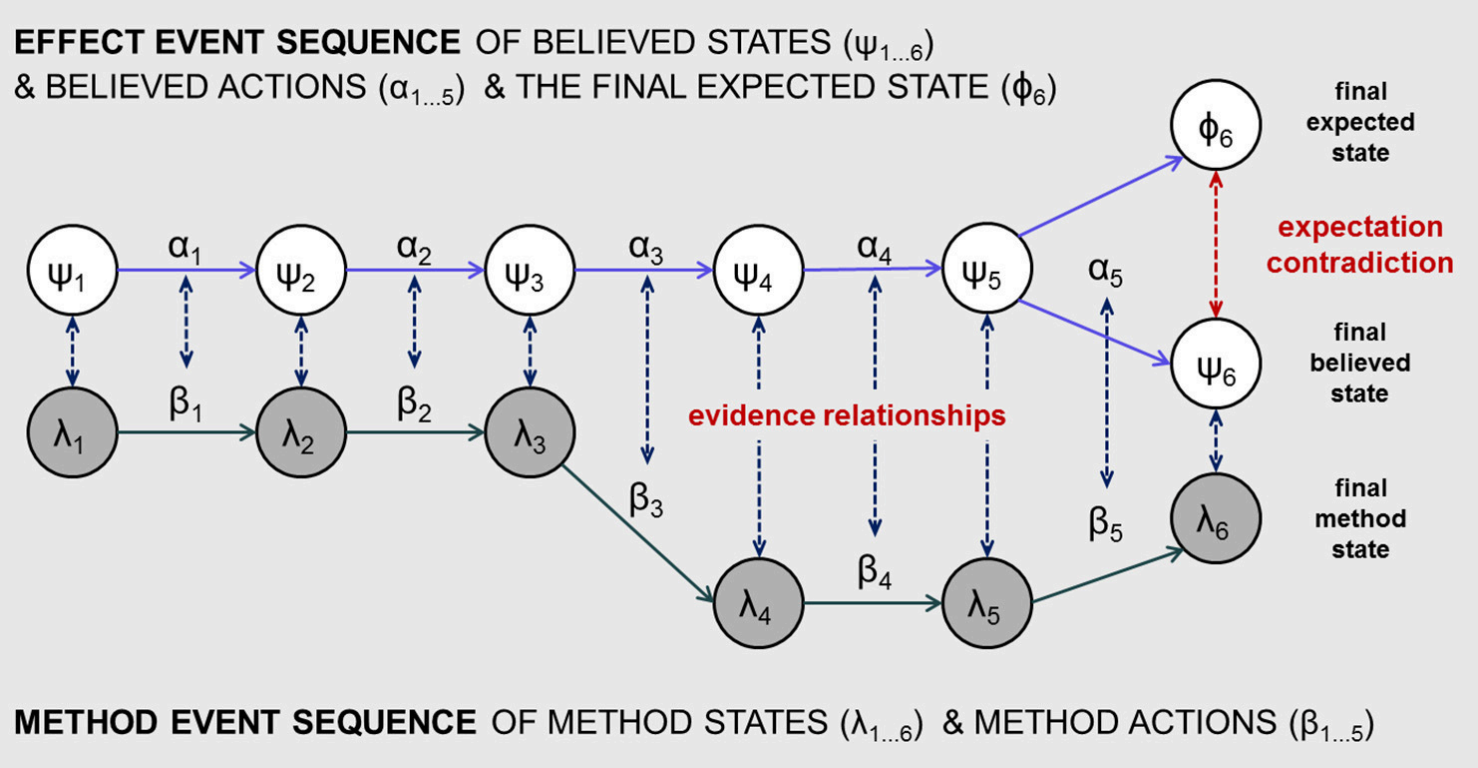
**A general model of a simple trick's event structure showing two parallel event sequences: an effect event sequence, that is believed to have occurred by the spectator, and a method event sequence, understood by the magician to have occurred**. The figure illustrates the particular case of there being six discrete time episodes, while in general there could any number greater than one. Impossibility is experienced at the end of the trick when three final states are distinguished: an *expected state* (supported by memory of the event history) which is in contradiction with a *believed state* (supported by current perception) and a *method state* of how the magician understands the final situation. The diagram also depicts a common (but not universal) pattern of evidence relationships in which stronger evidence exists at the beginning and end of the sequences (depicted as shorter evidence relationship arrows) and weaker evidence exists in the middle of the sequences (depicted as longer evidence relationship arrows). This common pattern is discussed in the text.

Drawing on these broad principles of magic trick construction, we now attempt to sketch the beginnings of a more formal account of how a belief in an impossible event is constructed. This offers a more precise understanding, although inevitably it sacrifices the richness and depth of the magicians' instructive principles. In the following, we first develop a definition of impossibility which allows us to better articulate the question that our account seeks to address. We then conceptualize how the experience of impossibility might arise. As Figure 1 shows, our account focuses on the relationship between two parallel event sequences that run over the course of a trick's performance: an *effect sequence* of events intended for the spectator to perceive and believe and which culminate in the experience of impossibility; and a *method sequence* of events known about by the magician, including states and actions kept secret from the spectator, which provides a non-magical description of what happens during the trick.

### Impossibility as an expectation contradiction in the effect sequence of events

We start with the view that impossibility arises as a conflict between a perception-supported believed state for a current situation, let's call it ψ, and an expected state Φ for that same situation; for example, a conflict between a currently perceived rabbit in a hat, coupled with an expectation that the hat is empty. For such conflicts to achieve a sense of impossibility depends on two things. Firstly, states ψ and Φ must be negations of each other, implying that they cannot both be true. The hat cannot have a rabbit in it and be empty. Secondly, the expected state Φ must be supported by a memory of having perceived and believed a history of past states (ψ_1…_ψ_n_) commencing from the trick's beginning (time t_1_) and leading to the end of the trick (time t_n_), and a related sequence of actions (α_1_ …α_n−1_) that together would normally lead to the expected state Φ. Continuing the example, the spectator of the rabbit in the hat must have a memory of perceiving and believing in a series of states and actions from time t_1_ onwards, which support the expectation of the hat being currently empty at time t_n_. This history of believed states and actions constitutes the effect sequence of the trick.

Here, and later in the article, we will capture these ideas informally using propositional dynamic logic, a formalism that was first defined by Fischer and Ladner (1979), and has been widely used in the analysis of computer programs. We refrain from a complete definition of that logic, but rather use the elements that are needed in a descriptive way to identify the key propositions being made. However, a full formal account in this logic could also be given.

In this account, then, impossibility exists as a contradiction between a perception-supported belief ψ and a memory-supported belief Φ. The question that we seek to address through the following analysis is how does such a contradiction arise? Why does an agent retain both beliefs when normal sense-making mechanisms might be expected to discount the weaker belief in favor of the stronger, or to discount both? How is it that a cognitive agent, in this case a spectator, comes to hold two inconsistent beliefs?

In practice, the impossibility condition is reached in different ways in different conjuring tricks. But typically, and in line with previous accounts of conjuring, it depends on misperceptions and misattentions of the trick's events. However, what our constructional emphasis asserts is that reaching the impossibility condition also depends on a carefully crafted history of events, including both their veridical and false aspects. It is how this history of veridical and false elements are constructed within the larger sequence of events that is critical to reaching the condition of impossibility.

**Impossibility as an Expectation Contradiction in the Effect Sequence of Events**

We define the condition in which a spectator experiences a situation to be impossible as:

          impossible(S, ψ) = believes(S, ψ) & expects(S, Φ) & ψ = ¬ Φ

where, S denotes a spectator

          ψ denotes the currently believed state of the situation

          Φ denotes the currently expected state of the situation

          ψ = ¬ Φ indicates that not both can be true

To identify what gives rise to the belief and what gives rise to the expectation, we first declare a history of 1 .. n states and actions which lead to the impossible situation comprising a final believed state, ψ_n_, and a final expected state, Φ_n._

Support for the final believed state comes directly from perceptual evidence:

          believes(S, ψ_n_) ← perceives(S, λ_n_)

Where,

          ← denotes that the perception *implies* the belief

          λ stands for the “actual” situation, as explained in the section “The Method Sequence of Events”.

Support for the memory-based expectation comes from:

          expects(S, Φ_n_) ← believes(S, believed(S, ψ_1…_ψ_n−1_))

                                   & believes(S, DONE(α_1_…α_n−1_))

                                   & believes(S, support(Φ_n_, ψ_1…_ψ_n−1_, α_1_…α_n−1_))

This asserts that S expects Φ_n_ to be true because she believes that she previously believed in the sequence of states ψ_1…_ψ_n−1_ before arriving at the current state ψ_n_; and S also believes that the sequence of actions α_1_…α_n−1_ has been done; and that normally by performing action α_1_ one gets from ψ_1_ to ψ_2_ and so on, and that the last action α_n−1_ would normally lead from ψ_n−1_ to Φ_n_.

### The method sequence of events: “actual” states and actions

While the impossibility condition has been defined chiefly in terms of two states, a perceived situation ψ and an expected situation Φ, a third state is also relevant. We will call this the method state, denoted as λ, referring to the state that is believed to hold true by another agent such as the magician who knows how the trick is done. The method state might informally be called the “actual situation” in the sense that it renders the trick as something possible rather than impossible. For the trick to work, and for impossibility to be achieved, it is necessary that λ is taken by spectators to be ψ. Extending this further, we can conceive of λ as the end point of a second sequence of events which define how the magician understands the full history of the trick. As shown in Figure 1, this method sequence can be conceived as a parallel sequence of method states (λ_1…_λ_n_) and method actions (β_1_…β_n−1_).

On reaching the condition of impossibility, because of its inherent contradiction, the spectator will scrutinize the situation in search of new evidence to modify or discount ψ or Φ or both, so as to render the situation as being possible. The perceptually-based belief in ψ can be scrutinized by further examination of the current situation, while belief in the expected state Φ can be scrutinized only through reconsideration of remembered events. For the final perception-based belief ψ_n,_ scrutiny means asking the question how did ψ_n−1_ become ψ_n_ under action α_n−1_? How did the empty hat become the hat with a rabbit inside, just by tapping it with a wand? This might entail searching for a hidden method state λ_n_ which is close to the expected state Φ_n_ but just appears to be ψ_n_. In our example, the spectator might first check to see that it is a real rabbit and not a fluffy toy that is easily folded away. But this search is typically fruitless because the final method state λ_n_ is closer to the perceived state ψ_n_ and the two are not easily discriminable, and both are very different to the final expected state Φ_n_. In our example, both λ_n_ and ψ_n_ involve a real live rabbit and this is the seemingly impossible element, because it is irreconcilable with the firm expectation that the hat should still be empty (Φ_n_). The question becomes how does this contradictory pattern of beliefs come about?

### Evidence relationships between the effect and method sequences

Figure 1 depicts how the spectator typically reaches this experience of impossibility through a sequence of method states and actions that secretly takes the actual situation away from the effect sequence during the course of the trick. That is, the unusual final situation of the trick comes about through the parallel and incremental construction of two contradictory outcomes: the effect sequence builds the spectator's expectation in Φ_n_, and the method sequence builds a different final state λ_n_ which is readily perceived by the spectator as the contradictory state ψ_n_.

This brings us to the question of how the method events remain hidden and unsuspected during the performance of the trick. At each moment, a method state λ gives off evidence that leads to a corresponding believed state ψ. Similarly, each method action β gives off evidence that leads to a corresponding believed action α. Figure 1 depicts this as a series of evidence relationships between each pair of corresponding states and actions in the effect and method sequences. We will now identify four important kinds of evidence relationship that might hold (summarized in Table 1), although there may be others. These form a pivotal part of our account. Each evidence relationship defines how the method state λ is taken to provide evidence for the corresponding belief in ψ, and likewise for actions.

**Table 1:** **Four types of evidence relationship between effect events and method events**.

	**Relationship between corresponding elements in the effect and method event sequences**	**Actions which might reveal inconsistencies between corresponding elements of the effect and method event sequences**
**Similarity**	Appearing similar but with small inconsistencies in the available perceptual evidence. (e.g., Effect state: a 10 of diamonds is shown; Method state: the card has one pip missing.)	Shifting attention to discrepancies between method and effect, or scrutinizing relevant states and actions more closely. (e.g., Counting the pips on the card.)
**Perceptual equivalence**	Inconsistencies exist but are not apparent in the available perceptual evidence, though they are apparent in aspects of the situation that are currently hidden. (e.g., Effect state: a card believed to be the 10 of diamonds is face down on the table; Method state: the 10 of clubs is face down on the table.)	Intervening in the situation to gain new perceptual evidence that reveals an inconsistency between method and effect. (e.g., Turning the card over to see its face.)
**Structural equivalence**	Inconsistencies exist but are not apparent through any evidence that could be extracted from the current situation, though they are apparent in comparisons to earlier states in the event sequence. (e.g., Effect state: A card that was previously on the top of the pack is now face up on the table; Method state: The card on the table was previously second in the pack.)	Comparing aspects of the current state with remembered previous states in the event sequence. (e.g., Noticing a blemish on the tabled card, and remembering that the previously top card did not have this blemish.)
**Congruence**	No inconsistencies exist. (e.g., Effect state: The 10 of diamonds lies face up on the table; Method state: The 10 of diamonds lies face up on the table.)	No action can reveal an inconsistency.

Although the examples given in this section all relate to states, the four evidence relationships also apply to actions. Further, they are ordered in their level of strength to withstand scrutiny: from similarity (weakest), through perceptual equivalence, structural equivalence, to congruence (strongest). As we explore in the next section, this strength bears on the role they typically play in the design of a trick's event structure and how they contribute to its impossible outcome.

#### Similarity

This relationship holds when there is at least one small inconsistency between the method state λ and the believed state ψ. An inconsistency means that a proposition entailed by one state is negated by a proposition entailed by the other state, and therefore λ and ψ cannot both be true. Under *similarity*, inconsistencies are apparent in the perceptual evidence given off by λ and so could be detected through greater perceptual scrutiny of the situation. But in practice, because the inconsistencies are small, they likely go unnoticed by the spectator who continues to accept the believed state ψ as holding true. For example, suppose the spectator believes state ψ, the 10 of diamonds is lying face up on the table, while the magician knows of a corresponding method state λ in which the card on the table is specially faked to resemble the 10 diamonds with the label “10” but only 9 pips. The spectator does not notice this difference, though closer scrutiny (counting the pips) would reveal the inconsistency between ψ and λ.

#### Perceptual equivalence

This also concerns cases when there are inconsistencies between λ and ψ. But now the consistencies are not visible because the available perceptual evidence given off by λ is identical to that which would be given off by ψ. Under *perceptual equivalence*, the inconsistencies between λ and ψ could be detected by intervening in the situation to obtain further perceptual evidence. For example, the spectator believes ψ, that the queen of diamonds is lying face down on the table, while the magician knows λ, that the two of clubs is lying face down on the table. No amount of scrutiny of the available perceptual evidence would reveal an inconsistency between ψ and λ. But obtaining new perceptual evidence, for example turning the card over, would reveal a difference.

#### Structural equivalence

Again this applies to cases for which inconsistencies exist between λ and ψ. However now, not only is the available perceptual evidence given off by λ identical to that for ψ, but also no amount of intervention in the current situation to gain further perceptual evidence could reveal an inconsistency between them. Under *structural equivalence*, the inconsistencies that exist can be revealed only by comparing the current situation against memories of past states. For example, the spectator believes state ψ, that the face down card on the table is whatever card was on the top of the pack at an earlier time, while the magician knows that the same tabled card is whatever card was on the bottom of the pack at that earlier time. No amount perceptual scrutiny or intervention, such as turning the card face up, or change of attentional focus could expose an inconsistency between λ and ψ. However, the inconsistency could be revealed by remembering what card was on the top of the pack earlier and finding a way to compare it with the tabled card. For example, the spectator might remember that the previous top card had a blemish that the tabled card does not have.

#### Congruence

The evidence relationship of *congruence* is different to the others in that it holds when there are no inconsistencies between the situation as believed by the spectator, ψ, and that known by the magician, λ. The two states may entail different propositions, but no proposition entailed by one is inconsistent with any proposition entailed by the other; therefore, ψ and λ could both be true. No further collection or scrutiny of perceptual or memorial evidence, even if perfect, could reveal the two situations as being inconsistent. For example, the spectator believes that the face down card is the four of clubs, and the magician knows that the face down card is the four of clubs. The magician and the spectator may know or believe various other things about the situation, but none of these are inconsistent with the four of clubs being face down on the table.

## An application of the concepts to Martin Gardner's *Turnabout*

**Figure 2:**
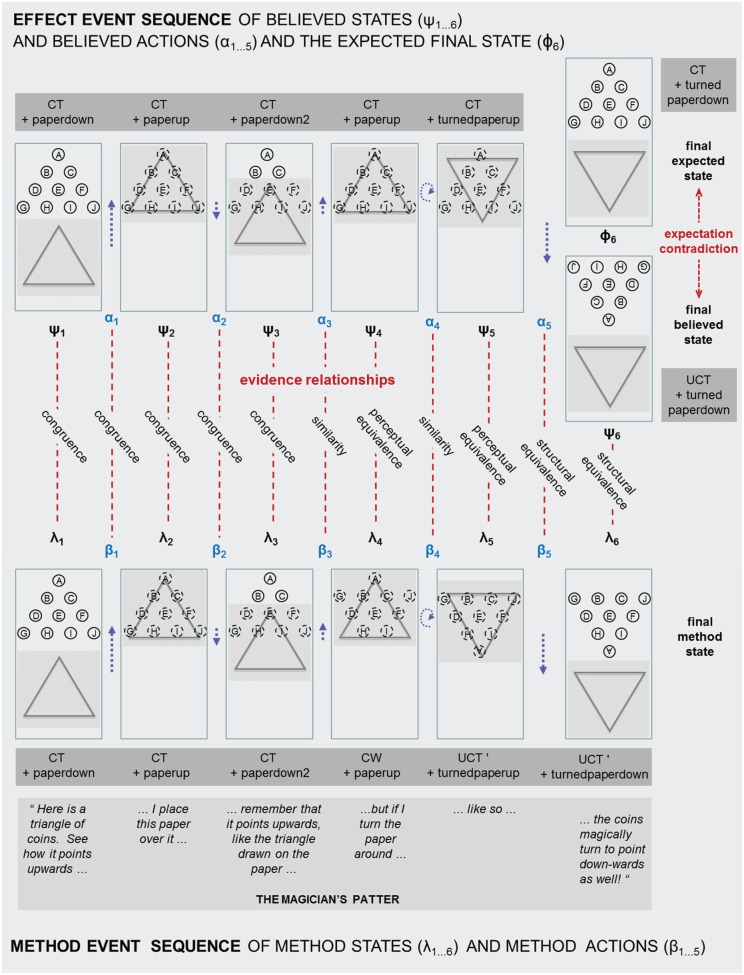
**An analysis of the trick ***Turnabout*** which shows it as an instantiation of the general model shown in Figure 1**.

To illustrate the application of the concepts developed, we now present an analysis of a particular magic trick, *Turnabout* (Fulves, 1977) invented by the popular mathematician Martin Gardner. *Turnabout* is chosen an example of a simple trick in that it presents a single effect using unfaked props, and has what magicians call a clean entry and a clean ending, meaning that everything is free for inspection by a spectator at the beginning and at the end. Even this simple trick will be seen to rest on a carefully crafted pattern of beliefs. *Turnabout* also has a sufficiently complex trajectory of hidden events to make it a valuable illustration of the account. In the following, we first present a purely textual description of *Turnabout*, followed by a more detailed analysis. Figure 2 serves as an illustration of both the informal description and the application of the formal concepts. A video demonstrating *Turnabout* is also included as supplementary material for this article (Video 1).

### An informal description of *Turnabout*

*Turnabout* is performed on a flat surface using 10 identical coins and a sheet of paper approximately 25 cm square. The effect is that a triangular array of coins magically transforms itself to point in the opposite direction. This occurs as an apparent sympathetic reaction to a piece of paper being placed over the triangle and turned through 180°. In the version described here, the sheet of paper has an equilateral triangle drawn on one side to mirror the coins and to mark its direction of facing.

Figure 2 shows *Turnabout* in six steps with illustrative patter. Assume that the magician and a spectator face each other across a table on which the trick is performed.

Step 1. The magician places 10 coins on the table in the formation of a triangle. The magician points out that the apex of the coin triangle points upwards toward the spectator.
Step 2. The magician places the paper over the coin triangle, covering it completely. The magician points out that the triangle drawn on the paper points in the same direction as the coin triangle.
Step 3. The magician pulls back the paper cover, enough to reveal the top 2 rows of the coin triangle as a reminder that it points towards the spectator and that it points in the same direction as the triangle drawn on the paper.
Step 4. The magician moves the paper forward again to cover the coin triangle.
Step 5. The magician then rotates the paper through 180 degrees, so that it still covers the coin triangle but is reversed in orientation and the triangle drawn on the paper now points down and away from the spectator.
Step 6. The magician slides back the paper to reveal that the coin triangle has also magically rotated through 180 degrees, so that its apex now also points down and away from the spectator!

The secret of the method is that really only three coins are moved, this being sufficient to create a new triangle that points in the opposite direction. The movement of the three coins is achieved in two steps. At step 4, as the coin triangle is re-covered, two coins are slid forward with the paper (coins G and J in Figure 2). Later, at step 5, when the card is rotated, the single coin A, at the apex of the coin triangle, is moved round to the other side of the configuration as the paper is rotated.

### A detailed analysis of *Turnabout*

We now present a more fine-grained description of *Turnabout* to illustrate the concepts developed earlier for the construction of impossibility. Figure 2 shows this interpretation as an instance of the general model depicted in Figure 1. For each step of the trick, we give a detailed qualitative account that operationalizes the concepts, with related logical propositions shown in accompanying boxes. Although these propositions are necessarily incomplete, and are therefore descriptive in form, their value is in distilling the most essential concepts and relationships.

To frame the account, we describe a world in which the magic trick occurs, including a magician (M), a spectator (S) and various objects and actions to be defined. The world is described as moving through 6 moments in time, equivalent to the 6 steps described. The aim is to provide a description of how the experience of impossibility is reached by the final step 6, and to show how it is constructed across the events of the previous steps, so demonstrating the *principle of the whole* as described earlier. The account traces two parallel state paths: an effect sequence, of what S is led to believe, and a method sequence, of what M understands as “actually” taking place. The effect sequence is made up of believed states (ψ) and believed actions (α), while the method sequence comprises a corresponding set of method states (λ) and method actions (β). All of these states and actions refer to physical objects and events in the world of the trick. For each step of the trick, various propositions are developed to describe how S comes to develop her beliefs (shown in accompanying boxes for each of the following sections).

#### World at time 1: state 1

The coin triangle (CT, as labelled in the accompanying formalisms) is presented with the paper cover, in a position down below the coins (paperdown), and M draws the attention of the spectator (S) to them through patter (see Figure 2) or gesture, or simply by bringing them into the zone of performance. It is only at this time 1 and later at the final time 6, that S is able to perceive the whole situation comprising all the coins and the paper cover. S therefore forms a belief about CT and the paper that is fully supported by perception and which is underpinned by a relationship of congruence with the method state. This belief encompasses the overall configuration of CT as pointing upwards, and also the position of the paper cover and its matching upwards orientation as shown by the triangle drawn on it. The *principles of naturalness and clarity* are vital here, and indeed throughout the trick, to avoid constant suspicion that other actions and objects are at play; though for simplicity we will take them as assumed and do not refer to them explicitly.

Another important aspect of the **world at time 1**, relating to the *principle of focus*, is that there are many details that are available to be perceived, but which S will not focus on because they are not deemed relevant to understanding the situation. Significantly, focus will be placed on CT, the paper cover and their overall orientations, and they become part of the believed state. But individuating details about each coin will not be the subject of focus; such as their position within the triangle and their orientation, or distinguishing shininess or blemishes. This lack of focus on such distinguishing details makes the coins substitutable for each other, a point we return to later.

**World at time 1**

States

       method state λ_1_ entails the following propositions:

       CT {Meaning “*There is a coin triangle of 10 coins with a given overall configuration and overall orientation of pointing upwards”*.}

           & paperdown {Meaning “*There is a piece of paper in a position down below the coins and bearing a drawing of a triangle which also has an orientation of pointing upwards”*.}

           & position(coinA, p1 … coinJ, p10) {Meaning “*CoinA is at position p1 within CT*,” etc.}

           & orientation(coinA, o1 … coinJ, p10) {Meaning “*CoinA is at orientation o1,”* etc.}

       believed state ψ_1_ entails the following propositions:

           CT & paperdown

Support for the believed state

            believes(S, ψ_1_) ← perceives(S, λ_1_) & focuses (S, CT & paperdown) & congruent(S, ψ_1_, λ_1_)

This asserts that S perceives the method state λ_1_, i.e., the situation as M understands it to be true; and S focuses attention on CT and paperdown, but not on the position and orientation of individual coins; and because ψ_1_ and λ_1_ are congruent at time 1, this leads S to believe in ψ_1._

#### World at time 2: action 1 and state 2

The first method action, or “actual” action, of M is to slide the paper up into a position (paperup) where it covers and thereby hides CT entirely. The whole situation is no longer in view, and will remain partly obscured until the final state 6 of the trick. Therefore the continued belief in CT now rests partly on the expectation for it, and partly on the perception of visible things, still underwritten by an evidence relationship of congruence. This mixture of expectation and perception relates to *the principle of blurring perception and inference*. The expectation rests on S believing that the action of sliding up the paper has been done and that it has not altered the previously believed existence of CT. S finds this situation normal and non-magical because there is mutual confirmation between what is believed and what is expected based on the history of the previous state and action.

**World at time 2**

States and actions

          method action β_1_ and believed action α_1_ both entail: slideup(M, paperdown, CT)

          method state λ_2_ and believed state ψ_2_ both entail: CT & paperup

          ψ_1_ → [α_1_] ψ_2_ {Meaning that the action α_1_ leads from ψ_1_ to ψ_2;_ from previous time 1, ψ_1_ entails: CT & paperdown.}

As for time 1, the method state is also likely to entail other propositions about individual coins, but we omit these for simplicity in the remainder of the analysis.

Spectator experience

S experiences the situation as normal because the belief and expectation for the current state are consistent:

          confirmation(S, believes(S, ψ_2_), expects(S, ψ_2_))

Support for the expectation

          expects(S, ψ_2_) ← believed(S, ψ_1_) & believes(S, DONE(α_1_)) & believes(S, ψ_1_ → [α_1_] ψ_2_)

where,

          believes(S, DONE(α_1_)) ← perceived(S, β_1_) & congruent(S, α_1,_β_1_)

This asserts: that S expects ψ_2_ because she remembers believing in ψ_1_; and also she believes that action α_1_ has been done; and that it changes ψ_1_ into ψ_2_; and she believes that action was done because she previously perceived the method action β_1,_ that M understands to have happened; which is congruent with α_1_ at time 2.

Support for the believed state

          believes(S, ψ_2_) ← perceives(S, visible(S, λ_2,_ paperup)) & congruent(S, ψ_2,_λ_2_) & expects(S, ψ_2_)

This asserts that S believes in ψ_2_ through a combination of expectation and perception: because she expects ψ_2_ to be true for the reasons given above; and she perceives the visible part of situation λ_2_ i.e., the paper in the up position; and λ_2_ is congruent with ψ_2_.

#### World at time 3: action 2 and state 3

The next step draws on the *principle of the incidental* by introducing an interlude to the main plot which might be presented by M as an afterthought to confirm or “reinforce” (Lamont and Wiseman, 1999) what S already believes about the existence of CT. Having established CT and covered it with the paper, M now partly slides back the paper (slidedown2) to a new position (paperdown2) where it reveals the top 2 rows of coins (CTtop2rows) but still covers the bottom two rows. This is done ostensibly to remind S that the coin triangle points upwards and in the same direction as the triangle drawn on the paper. As before, the believed situation is produced by a mixture of expectation and perception. The result is experienced by S as normal, because the expectation based on the event history so far is consistent with the visible perceptual evidence. Again, this is underwritten by the believed events and method events being congruent.

**World at time 3**

States and actions

          method action β_2_ and believed action α_2_ both entail: slidedown2(M, paperup, CT) {Meaning to slide the paper down just 2 rows of coins.}

          method state λ_3_ and believed state ψ_3_ both entail: CT & paperdown2

          ψ_2_ → [α_2_] ψ_3_ {From previous time 2, ψ_2_ entails: CT & paperup.}

Spectator experience

          confirmation(S, believes(S, ψ_3_) & expects(S, ψ_3_))

Again, S experiences this situation as normal because the current believed state and expected state are consistent.

Support for the expectation

This is the same as that for time 2, except that the time is one step forward (i.e., ψ_3_ replacesψ_2_, and so on).

Support for the believed state

          believes(S, ψ_3_) ← perceives(S, visible(S, λ_3,_ paperdown2 & CTtop2rows)) & focus(S, paperdown2 & CTtop2rows) & congruent(S, ψ_3,_λ_3_) & expects(S, ψ_3_))

This asserts a form of support for the current belief based on an evidence relationship of congruence, like that at time 2 as a mixture of perception and expectation, except additional support for ψ_3_ comes from the now visible top two rows of CT; and attention is again focused on the overall configuration of CT rather than on individual coins.

#### World at time 4: action 3 and state 4

At time 4, the believed action of M sliding the paper back up over the whole coin triangle (slideup2) reverses the previous action of time 3. Significantly, however, the method action at time 4, although similar to the believed action, is different in that it includes the secret and hidden movement of two coins (G and J, see Figure 2) from the outer ends of row 4, at the base of CT, up to row 2. This forms a new configuration of coins that we will call CW because it is no longer a triangle but resembles the letter “W.” This first secret movement of the trick has ongoing consequences for the evidence relationships between believed and method states. Unlike the simple congruence relationship that has held so far, the method action, of moving the paper up two rows plus secretly moving coins G and J, introduces an inconsistency between effect and method, and exhibits only a similarity relationship with the believed action of moving just the paper back up to cover the coins. They are similar in that the action of moving the paper and the coins up, is likely to be slightly, yet visibly, different to the simple action of moving the paper alone. The believed action could therefore be discredited from the perceptual evidence, because it is subtly different from the method action, but this inconsistency is unlikely to be noticed in practice.

Once the action has been taken, and CW has been formed, the method state now deviates from that which S believes to be true. S believes that CT is still intact, based on her belief that moving the paper up does not change anything except for CT becoming not visible. What is especially important here, is that the believed and method states now have a stronger evidence relationship than similarity, and are now perceptually equivalent. This means that the inconsistency between them is not apparent in the available perceptual evidence, although it could be revealed if the physical objects were investigated; in this case, if the paper was removed.

From M's point of view at time 4, the trick has reached its most vulnerable condition, because the believed and method states are highly inconsistent (CT vs. CW). The relationship of perceptual equivalence between them provides a strong enough protection against detection, provided that the procedure of the trick soon continues on beyond this state. Lingering in state 4, would allow S to question her belief about the continued existence of the currently hidden CT. Despite the discrepancies between the effect and method sequences in the world at time 4, S will continue to regard it as normal and non-magical because there is still confirmation between what is expected and what is believed to be the case.

**World at time 4**

States and actions

          method action β_3_ entails: slideup2(M, paperdown2 & coins(G, J), CT)

          believed action α_3_ entails: slideup2(M, paperdown2, CT)

          method state λ_4_ entails: CW & paperup {CW refers to the coins in a “W” configuration as shown in Figure 2.}

          believed state ψ_4_ entails: CT & paperup

          ψ_3_ → [α_3_] ψ_4_ {From previous time 3, ψ_3_ entails: CT & paperup2.}

Spectator experience

          confirmation(S, believes(S, ψ_4_) & expects(S, ψ_4_))

As before, S experiences this situation as normal because the current believed state and expected state are consistent.

Support for the (now false) expectation

          expects(S, ψ_4_) ← believed(S, ψ_3_) & believes (S, DONE(α_3_)) & believes(S, ψ_3_ → [α_3_] ψ_4_)

Where,

          believes(S, DONE(α_3_)) ← perceived(S, β_3_) & similar(S, α_3,_β_3_)

          similar(S, α_3,_β_3_) means: approximation(perceptual_evidence(S, α_3_), perceptual_evidence(S, β_3_))

This asserts that the expectation in ψ_4_ forms for the same reason as in earlier times, but now rests on the incorrect belief that α_3_ was done based on having perceived β_3_ which is similar to α_3_.

Support for the (now false) believed state

          believes(S, ψ_4_) ← perceives(S, visible(S, λ_4,_ paperup)) & perceptually_equivalent(S, ψ_4,_λ_4_) & expects(S, ψ_4_)

Where,

          perceptually_equivalent(S, ψ_4_, λ_4_) means: perceptual_evidence(S, ψ_4_) = perceptual_evidence(S, λ_4_)

Asserting that support for the belief in ψ_4_ comes from a mixture of perception, of the visible aspects of the situation, and expectation; combined with perceptual equivalence between ψ_4_ and λ_4_.

#### World at time 5: action 4 and state 5

Action 4 is the turning of the paper cover through 180° so that it now points downwards but is still in the up position covering the coins (turnedpaperup). It creates the moment when the trick moves beyond the preparation of the objects and becomes an action that is later purported to have a magical effect. As at time 3, the method action also contains a secret hidden movement, carrying coin A from the top of CW to the bottom and reversing the coin's orientation, so creating an upside-down coin triangle that we will call UCT′ (the significance of its configuration will be described in the next section).

The believed action of turning the paper around, over the top of CT, has an evidence relationship of similarity with the method action of turning the paper over CW plus the added movement of coin A. These actions are only similar to each other, as opposed to be being perceptually equivalent, for two reasons: (i) the action of carrying coin A with the paper is slightly different to the action it simulates, and (ii) as the paper turns, the coins underneath are likely to “flash”, meaning they become briefly visible to S who could in principle see that they are not positioned consistently with CT's configuration. Although similarity is the weakest evidence relationship, S will likely not notice these inconsistencies because they occur very briefly during the turn movement.

In contrast, the resulting method state at time 5 is available for greater scrutiny because it is static and persists for a longer duration. What is critical in the trick's construction, is that there is now a stronger evidence relationship of perceptual equivalence. That is, the perceptual evidence given off by the covered UCT′ is the same as that which would be produced by the covered CT. A small qualification is that UCT′ is actually one row of coins lower than the original CT, so we are assuming that the paper is large enough that its position does not need to be different in the two situations. Again, despite the growing inconsistencies between the effect and method sequences, S still finds the believed state as being normal and consistent with what they expect. As at earlier times of the trick, S continues to believe in CT even though it is not visible under cover of the paper.

**World at time 5**

States and actions

          believed action α_4_ entails: turn(M, paperup, CT)

          method action β_4_ entails: turn(M, paperup & coinA, CW)

          believed state ψ_5_ entails: CT & turnedpaperup {Meaning the paper turned downwards but still in the up position over the coins.}

          method state λ_5_ entails: UCT′ & turnedpaperup

          ψ_4_ → [α_4_] ψ_5_ {From previous time 4, ψ_4_ entails: CT & paperup.}

Spectator experience

          confirmation(S, believes(S, ψ_5_) & expects(S, ψ_5_))

S continues to experience the situation as normal because the perception-supported belief and expectation are consistent.

Support for the (false) believed state and the (false) expected state

These are both supported in the same way as for time 4, except that now time is one step forward (i.e., ψ_5_ replaces ψ_4_, and so on).

#### World at time 6: action 5 and the final state 6

Finally the trick reaches its climax through the method action 5 of sliding down the previously turned paper (slidedown) to a position below the coins (turnedpaperdown). This reveals the impossible event: the coin triangle has magically turned upside-down in sympathy with the preceding turning of the paper. The experience of impossibility rests on two things being true. Firstly, there is a negation between the expected state of an upwards-pointing coin triangle CT, and the perceived state of the coins arranged as a downwards-pointing or upside-down triangle that we will call UCT. That is, it is not possible for both CT and UCT to be true. Secondly, there is strong memory-based support for the expectation of CT which in some sense matches the contradictory perceptual support for UCT.

Faced with the final experience of an impossible event, spectators will scrutinize their perceptual and memorial evidence more closely in an attempt to resolve the contradiction between the perceived UCT and the expected CT. What is critically significant for the success of the trick, at this final state 6, is that the evidence relationships are now strong. The relationship between the believed state and the method state presents a relatively complex situation. Let's assume that S believes the perceived upside-down coin triangle, UCT, was created by rotating the original CT through 180°; this assumption is reflected in the marking of coins in the effect sequence of believed states in Figure 2. In reality, the actual arrangement of the coins is something quite different, that we have called UCT′, which results from the secret method actions of sliding up coins G and J and then moving coin A to bottom of the configuration and reversing its orientation.

The result is that the believed and method states, at this final magical moment, have now taken on a relationship that is stronger than similiarity and perceptual equivalence, and achieved the status of structural equivalence. That is, the inconsistencies between UCT and UCT′ are not identifiable in the presently available perceptual evidence, and further they are not identifiable in any evidence that might be discoverable through rearranging the objects or shifting the focus of attention. Yet UCT and UCT′ fall short of being congruent, because they have inconsistencies that could be identified by comparison back to the details of previously encountered states (particularly, states 1 and 3). Such comparisons would depend on remembering details of individual coins such as blemishes or particular orientations. However, such details, are extremely unlikely to be available in memory at the time of state 6. As noted, this is therefore a case of what magicians describe as “ending clean,” meaning that S is free to search or interrogate the situation because, without the required memories, no discrediting evidence can be discovered. The final believed action and method action are also structurally equivalent to each other because, although the sliding down of the paper is itself potentially congruent across the two situations, as the coins are revealed they gradually exhibit the potentially discriminable inconsistencies just described.

**World at time 6**

States and actions

          believed action α_5_ entails: slidedown(M, turnedpaperup, CT)

          method action β_5_ entails: slidedown(M, turnedpaperup, UCT′)

          expected state Φ_6_ entails: CT & turnedpaperdown {Meaning the paper turned to point downwards and in the down position below the coins.}

          believed state ψ_6_ entails: UCT & turnedpaperdown

          method state λ_6_ entails: UCT′ & turnedpaperdown

          ψ_5_ → [α_5_] Φ_6_ {From previous time 5, ψ_5_ entails: CT & turnedpaperup.}

Spectator experience

          impossible(S, believes(S, ψ_6_) & expects(S, Φ_6_) & ψ_6_ = ¬Φ_6_)

S experiences the situation as impossible because there is a contradiction between the current believed state and the expectation.

Support for the (false) expectation

          expects(S, Φ_6_) ← believed(S, ψ_5_) & believes(S, DONE(α_5_)) & believes(S, ψ_5_ → [α_5_] Φ_6_)

Where,

          believes(S, DONE(α_5_)) ← perceived(S, β_5_) & structurally_equivalent(S, α_5,_β_5_)

          structurally_equivalent(S, α_5_, β_5_) means: discoverable_evidence(S, α_5_) = discoverable_evidence(S, β_5_)

This asserts that the false final expectation comes about in the same way as earlier expectations, but now rests on believing that the preceding state ψ_5_ was true and that action α_5_ was done and that normally this should lead to ϕ_6_. And α_5_ is believed to have occurred because the method action β_5_ was perceived and it is structurally equivalent to α_5_.

Support for the contradictory final believed state ψ_6_ comes now purely from perception:

          believes(S, ψ_6_) ← perceives(S, λ_6_) & structurally_equivalent(S, ψ_6,_λ_6_)

Where,

          structurally_equivalent(S, ψ_6,_λ_6_) means: discoverable_evidence(S, ψ_6_) = discoverable_evidence(S, λ_6_)

Asserting that belief in ψ_6_ comes now purely from perception of the situation λ_6_, as M understands it, and the evidence relationship of structural equivalence between ψ_6_ and λ_6_.

## Observations and issues arising from the analysis of impossibility

Conjuring is a rich and sophisticated craft and its tricks are designed and performed to work at different levels of spectators' understanding. Our account has focused on just one level, the arrangement of a trick's events to construct a history of beliefs leading to the experience of impossibility. At the risk of reductionism, we have not considered how this co-exists with the higher narrative level of conjuring tricks that creates meaning and emotional affect for spectators, as stressed by many magicians (e.g., Sharpe, 1932; Burger and Neale, 2009). Most notably, we have defined the experience of impossibility as encountering a situation that produces a striking contradiction between a perception-supported believed state and a memory-supported expected state. For magicians, the associated emotional reaction of spectators is paramount, and they strive to achieve something akin to a “sense of wonder” as described by Rensink and Kuhn (2015). Much of the skill of a magician lies in avoiding spectators adopting what Kelley (1980) called a “problem-solving” mode, of searching for the “actual” method sequence of events, and instead enabling them to accept and enjoy the magical effect sequence on its own terms. In this way, spectators may momentarily experience the outcome of a trick as not simply an anomalous event, but more as something that suggests different possibilities in the laws of nature akin to people's belief in real magic (Subbotsky, 2010).

Nevertheless, we contend that such higher-level affective responses in conjuring rest on striking and unavoidable contradictions at the level of perception and cognition. Hence we offer the present analysis as an account of how conjuring tricks are constructed to produce outcomes that seem to be logically at odds with our expectations. Even at this level of analysis, some further qualifications of our account are needed. One is that we have not considered events which work as perceptual illusions. These underlie many tricks, for example the vanishing ball trick (Kuhn and Land, 2006), by exploiting hard-wired properties of visual perception to deliver up false percepts, the basis of which are not accessible to direct scrutiny and hence are said to be cognitively impenetrable (Pylyshyn, 1984). In contrast, the evidence relationships we have identified (similarity, perceptual equivalence, structural equivalence, and congruence) are all cognitively penetrable in that they are not hard-wired results but are susceptible to cognitive interrogation. Another simplification in our account is that we consider memory supported beliefs as correctly registering the information that was previously attended to, while often the impact of a trick rests on significant distortions in the way events are remembered, both in short-term memory and when the trick is recounted much later (Wiseman and Lamont, 1996).

Another important aspect of our account is its detailed focus on just one simple trick. We have described a common, but not universal, pattern in which evidence relationships are relatively strong at the beginning and end of the trick and weaker in the middle when the greater part of the secret work is done to separate the actual and believed situations. It should be noted that other successful tricks employ different patterns, and many end on effects that rely on weaker relationships of similarity or perceptual equivalence. Such tricks typically require an extra “clean up” phase to remove their vulnerability to discovery, often by moving swiftly on to the next trick. What we have shown in our account, therefore, is not a definitive pattern, but rather an illustration of a set of relevant concepts for interpreting the various ways that impossibility is constructed. Nor are these concepts intended to be exhaustive, for example there are likely to be other kinds of evidence relationship.

Notwithstanding these qualifications, we have attempted to demonstrate that the construction of impossibility in conjuring requires something more than isolated misperceived and/or misattended events. Although these are typically vital ingredients, impossible effects are created through the whole sequence of events making up a trick's performance, both veridical and false; an idea well-grounded in magicians' key instructional texts. To sketch the beginnings of a simple logical framework for how the experience of impossibility is constructed, we started with the notion of it as a contradiction between a perception-supported belief about a situation and a memory-supported expectation for the same situation. The experience is characterized by an inability to resolve the contradiction of believing in both of these states, despite them being in logical opposition to each other, because neither the final believed state nor the final expected state can be rejected in favor of the other.

Developing this further, and extending the analysis of Kelley (1980), we have proposed that the history of a trick's events can be understood as two parallel sequences: an effect sequence of believed states and actions, and a method sequence of “actual” or method states and actions. The sequence of method states λ_1_ to λ_n_ incrementally transforms an initial situation into one that gives rise to a believed state ψ_n_ that is in strong contradiction with the expected state Φ_n_ (as shown in Figures 1, 2). In contrast to the spectators' sense of a sudden magical and inexplicable state transformation, the method state gradually undergoes many smaller changes, each designed to remain undetected and unsuspected. In our account, then, the construction of impossibility is seen to be diffused across the trick's event history.

Based on this account, we will now propose three further principles related to the construction of impossibility that might be added to our initial set based on our reading of magicians' texts, comprising naturalness, the whole, clarity, focus, the incidental, blurring perception and inference, no-notice and early denial. The three further principles are not intended as being new to magicians, but rather they are so deeply implicit in their craft that they are typically not made explicit in instructional texts.

### The principle of equivalence

Our analysis of Martin Gardner's *Turnabout*, has illustrated what can be called the *principle of equivalence*, referring to the management of different kinds of evidence relationship over a trick's history. It was seen that each state of the method sequence gave off perceptual evidence to support a corresponding believed state within the effect sequence. Likewise for actions. We identified four kinds of evidence relationship that might hold for any pair of states or actions: *similarity* (the weakest) in which they appear similar but inconsistencies could be detected through greater scrutiny; *perceptual equivalence*, in which they give off identical perceptual evidence but inconsistencies could be revealed by intervening in the situation to get new evidence; *structural equivalence* in which they give off identical perceptual evidence but inconsistencies could be found through comparison with memories of earlier states; and finally *congruence* (the strongest) in which there are no inconsistencies between corresponding pairs of believed and method states or actions.

It has been seen how the impossible outcome depends on the careful design and performance of these evidence relationships over the course of the trick. Significantly, there is an alignment of evidence strength with the level of scrutiny to be faced. The construction of the trick is built around relatively strong evidence relationships, of congruence and structural equivalence, at its beginning and at its final impossible event. Both the beginning and end of the trick (state 1 and state 6) are times of high spectator scrutiny. The impossibility of the final event triggers the highest scrutiny, but the opening of the trick is also heavily scrutinized as the situation is first established. In contrast, the middle events of the trick are characterized by the weaker relationships of similarity and perceptual equivalence. However, these events face far lower scrutiny because they are non-magical and aligned with expectations that are built through the effect sequence. Hence, the trick is designed with strongest evidence meeting greatest scrutiny, and weakest evidence meeting weakest scrutiny. Also important is that the construction of the trick depends on the limits of recovering information from memory. While the impossible final event is subject to great perceptual scrutiny, as the spectator attempts to resolve its inherent contradiction, the weaker evidence of the trick's middle events cannot be subject to such scrutiny in retrospect and cannot be intervened in for more evidence.

### The principle of substitutable elements and the principle of stable occlusion

There are two further principles associated with our analysis that we have not yet discussed, and again they are deeply implicit in the magician's craft. They both express general properties of apparatus used by magicians that are not explicitly named in conjuring texts but which are ubiquitous and instrumental in supporting the construction of impossibility in the way described here. The first, that we call the *principle of substitutable elements*, is that magical apparatus typically contains repeating elements (cards, coins, cups, balls, rings, walls of cabinets) where one is not easily distinguishable from another in many situations. Even in 1584, Reginald Scot identified three types of magic “with balls, with cards and with money” (Dawes, 1979, p. 17), all of which support substitution. The trick *Turnabout* has been seen to rely on the spectator perceiving a false correspondence of coins between upwards-pointing and downwards-pointing coin triangles (see Figure 2). This is only possible because the spectator does not attend to the individuating features of each coin, such as orientation or blemishes, and hence they become substitutable for each other. The result is that the magically upside-down triangle of coins (UCT) is indistinguishable from, and hence structurally equivalent to, the actual final configuration (UCT′). In his analysis of magic in terms of causal attribution, Kelley drew a comparison between this substitution of elements in conjuring and apparent motion effects as in the phi phenomenon.

The second principle about magic apparatus, that we will call the *principle of stable occlusion*, concerns the way various aspects of a situation can be partially covered and uncovered from the spectator's view. A person is placed inside a box to be sawn in half, a rabbit appears from inside a hat, cards can be turned face down, balls placed under cups, and coins held in closed hands. Without objects or aspects of objects moving temporarily in and out of view, there is little scope to perform secret actions, or to suspend the moment when the results of secret actions are revealed. A critically important aspect, hence our reference to *stable* occlusion, is that an effective apparatus must be such that spectators have complete confidence that the concealed objects, or object parts, are not vulnerable to unseen changes: a face down card on an open table will retain its identity; a ball under a cup on a solid table cannot be secretly accessed. It is only when spectator are completely confident that a hidden thing cannot be changed, that they are astonished when it has.

In general, the principle of substitutable elements in apparatus supports the creation of structural equivalence between effect and method, because repeating elements (like coins, face-down cards, cups and balls) can be passed off as each other; with no form of detection other than comparing them against memories of earlier events. The principle of stable occlusion, on the other hand, supports the creation of perceptual equivalence, because the hidden parts of a situation can become discrepant from the believed state while the visible parts remain consistent.

## Conclusion

The experiences of impossibility created by magic tricks are unusual cognitions and emotions that require a different kind of explanation to those given for how events are misperceived or misattended. We have presented one approach to understanding the cognitive aspects of impossibility through an analysis of its logical form considered as a contradiction between an expected state and a believed state. For this sense of impossibility to persist depends on the contradiction remaining unresolvable. This in turn depends on strong perceptual evidence for the current believed state and equally strong memory-based support for the conflicting expected state. Our account offers an explanation for how this situation can be created through the constructional aspects of a conjuring trick, implying the way that its events are organized over the course of the whole performance. We have described how two event sequences run in parallel throughout—an effect sequence and a method sequence—and how the trick is carefully designed to manage what we have called the evidence relationships between them.

The logic-based account that we have presented is at an early stage, focussing on the most rudimentary aspects of a simple single-effect conjuring trick. It is a long way off capturing the many significant subtleties of conjuring, even within the perspective of cognitive belief formation; such as multiple effects within a routine, pretended failures, and double bluffs. Nevertheless, our account takes a first step by demonstrating that the impossible outcome of even the simplest of tricks depends on a carefully designed and performed history of events and beliefs.

## Author contributions

WS conducted the review of magicians' instructional text and led the development of the qualitative account. FD and LS developed the formalisms using propositional dynamic logic.

### Conflict of interest statement

The authors declare that the research was conducted in the absence of any commercial or financial relationships that could be construed as a potential conflict of interest.

## Supplementary material

Video 1 **Turnabout**.
